# Functional Metagenomics Reveals a New Catalytic Domain, the Metallo-β-Lactamase Superfamily Domain, Associated with Phytase Activity

**DOI:** 10.1128/mSphere.00167-19

**Published:** 2019-06-19

**Authors:** Genis Andrés Castillo Villamizar, Katrina Funkner, Heiko Nacke, Karolin Foerster, Rolf Daniel

**Affiliations:** a Department of Genomic and Applied Microbiology and Göttingen Genomics Laboratory, Institute of Microbiology and Genetics, Georg-August University of Göttingen, Göttingen, Germany; b Línea Tecnológica Biocorrosión, Corporación para la Investigación de la Corrosión C.I.C., Piedecuesta, Santander, Colombia; Martin Luther University of Halle-Wittenberg Institute of Biology/Microbiology

**Keywords:** β-lactamase, metallo-β-lactamases, phosphatase, phytase, phytate, soil metagenome

## Abstract

Inositol-6-phosphate, also known as phytic acid, is a phosphorus source that plays several important roles in the phosphorus cycle and in cell metabolism. The known characterized enzymes responsible for its degradation, the phytases, are mostly derived from cultured individual microorganisms. The catalytic signatures of phytases are restricted to the molecular domains of four protein superfamilies: histidine phosphatases, protein tyrosine phosphatases, the purple acid phosphatases and the β-propeller phosphatases. During function-based screening of previously generated forest soil metagenomic libraries for Escherichia coli clones conferring phytase activity, two positive clones harboring the plasmids pLP05 and pLP12 were detected. Analysis of the insert sequences revealed the absence of classic phosphatase/phytase signatures of the proteins deduced from the putative genes, but the genes *mblp01* (pLP05) and *mblp02* (pLP12) encoded putative metallo-β-lactamases (MBLs). Several MBL representatives are promiscuous proteins with phosphoesterase activity, but phytase activity was previously not reported. Both *mblp01* and *mblp02* were subcloned, expressed, and analyzed. Mblp01 and Mblp02 are members of the lactamase B2 family. Protein modeling showed that the closest structural homologue of both proteins was ZipD of E. coli. Mblp01 and Mblp02 showed activity toward the majority of the tested phosphorylated substrates, including phytate. The maximal enzyme activities were recorded for Mblp01 at 50°C under acidic conditions and for Mblp02 at 35°C and a neutral pH. In the presence of Cu^2+^ or SDS, the activities of Mblp01 and Mblp02 were strongly inhibited. Analyses of the minimal inhibitory concentrations of several β-lactam antibiotics revealed that recombinant E. coli cells carrying *mblp01* or *mblp02* showed reduced sensitivity toward β-lactam antibiotics.

**IMPORTANCE** Phytic acid is a phosphorus storage molecule in many plant tissues, a source of phosphorus alternative to phosphate rocks, but it can also be a problematic antinutrient. In comparison to other phosphorus sources, phytic acid exhibits reduced bioavailability. Additionally, it influences functions of secondary messengers and acts as antioxidant in tumor growth prevention. The enzymatic capability to process phytate has been reported for a limited number of protein families. This might be due to the almost exclusive use of proteins derived from individual microorganisms to analyze phytase activity. With such a restriction, the study of the complexity and diversity of the phytases remains incomplete. By using metagenome-derived samples, this study demonstrates the existence of phytase activity in one of the most promiscuous superfamilies, the metallo-β-lactamases. Our results increase the general knowledge on phytase diversity in environmental samples and could provide new avenues for the study and engineering of new biocatalysts.

## Abstract (precis)

Phytic acid is a phosphorus storage molecule in many plant tissues, a source of phosphorus alternative to phosphate rocks, but it can also be a problematic antinutrient. In comparison to other phosphorus sources, phytic acid exhibits reduced bioavailability. Additionally, it influences functions of secondary messengers and acts as antioxidant in tumor growth prevention. The enzymatic capability to process phytate has been reported for a limited number of protein families. This might be due to the almost exclusive use of proteins derived from individual microorganisms to analyze phytase activity. With such a restriction, the study of the complexity and diversity of the phytases remains incomplete. By using metagenome-derived samples, this study demonstrates the existence of phytase activity in one of the most promiscuous superfamilies, the metallo-β-lactamases. Our results increase the general knowledge on phytase diversity in environmental samples and could provide new avenues for the study and engineering of new biocatalysts.

## INTRODUCTION

Over the last 2 centuries, anthropogenic activities have significantly altered the global biogeochemical cycles of elements such as carbon (C), nitrogen (N), and phosphorus (P). With the rising global population, the P flow to the biosphere has been quadrupled. This cycle disruption is mainly due to the mining of P compounds for fertilizers. P sources such as phosphate rocks are not renewable, and the current resources are being rapidly depleted (1, 2). Consequently, a better understanding of the P cycle, and development of strategies and alternatives for P acquisition are of increasing importance.

Phytic acid, also known as inositol-6-phosphate or phytate in its salt form, represents an immense and almost unexploited reservoir of P that potentially could be utilized by plants, microorganisms, and animals (3). In humans, phytate and some of its degradation products have been related to metabolic effects, such as the prevention of kidney stone formation and a possible protection against diseases such as diabetes mellitus, atherosclerosis, coronary heart disease, and some types of cancer (4, 5). Phytate is the most abundant source of P in several types of cereals and grains that are commonly used to feed animals employed for large-scale production. However, monogastric animals such as swine, poultry, and fish cannot digest phytate efficiently due to the lack of phytases. This limited capacity of phytate processing has led to severe environmental problems: i.e., P eutrophication of water bodies (3, 6).

Since the cleavage of the phosphate residues from phytate requires phytases, the search for new phytases with novel and improved characteristics has been one focus of phosphatase research. Several phytases have been reported and characterized revealing the existence of different catalytic mechanisms to cleave phosphate groups from phytate. Some phytases are used commercially to reduce the impact of phytate accumulation. Nevertheless, the *in vivo* functions of phytate and several phytate hydrolysis mechanisms are not fully known (7).

Phytase activity has been reported to be restricted to only four classes of protein phosphatases with different catalytic mechanisms. The histidine acid phosphatases (HAP-phy) represent the most extensively studied class of phosphatases to which all commercially used phytases belong (8). Another group of phytases comprises the relatively recently described β-propeller phosphatases (BPP-phy), which exhibit no significant homology to any known phosphatases. Furthermore, representatives of purple acid phosphatases (PAP-phy), which are mostly found in plants, and the protein tyrosine phosphatases (PTPs-phy, or cysteine phytases) that are the main phytate-degrading enzymes of ruminant animals (9), are known to exhibit phytase activity. In addition, several phytases have been associated with microbial pathogenicity in different species, i.e., a type III effector protein with phytase activity has been described as a key factor for the pathogenicity of the plant pathogen *Xanthomonas*. Other phytases contribute to the virulence potency in human fungal pathogens such as Candida albicans (10–13). The vast majority of reported phytases are derived from a small culturable fraction of microorganisms, but recent reports implementing functional metagenomic approaches demonstrated the potential of environmental samples as source of novel phosphatases/phytases (14, 15).

In this study, two environmentally derived promiscuous enzymes (Mblp01 and Mblp02) exhibiting phytate-degrading activity and carrying the catalytic domain of metallo-β-lactamases (MBLs) were characterized. To our knowledge, this superfamily domain has never been associated with this type of activity before. MBLs catalyze the hydrolysis of a wide range of substrates such as β-lactam antibiotics, including penicillin and the latest generation of cephalosporins. Different members of this family also mediate glycosylase, lactonase, arylsulfatase, 5′-exonuclease or RNase activities. In addition, phosphoesterase activity has been reported (16).

## RESULTS

### Identification and sequence analysis of the novel phytase-encoding genes.

Two phosphatase/phytase-active E. coli clones harboring the recombinant plasmids pLP05 (2,496 bp) and pLP12 (5,578 bp) were identified by color change during the functional screening of soil metagenomic libraries using phytic acid as the sole P source (see Fig. S1 in the supplemental material) (14). Sequence analysis of the inserts did not reveal putative genes similar to known ones encoding phosphatase/phytase activity (see Fig. S2 and S3 in the supplemental material). Therefore, no phosphatase gene was initially assigned as responsible for the activity of the recombinant E. coli strains on indicator agar with phytic acid as a P source. However, the inserts pLP05 and pLP12 both hold the genes *mblp01* and *mblp02*, respectively. These genes encode proteins carrying the MBL fold. MBLs are considered to be a highly promiscuous superfamily that includes among others representatives of the arylsulfatases, RNases, and phosphoesterases (16, 17). Therefore, the genes *mblp01* and *mblp02* were individually subcloned and thereby generated recombinant E. coli strains, which were examined for color change as proof of activity on indicator agar. E. coli clones carrying the plasmid with the subcloned *mblp01* or *mblp02* gene developed the intense blue dye typical for phosphatase activity on indicator agar with phytic acid as the sole P source, demonstrating that *mblp01* and *mblp02* encode proteins with phosphatase/phytase activity.

10.1128/mSphere.00167-19.1 FIG S1 Appearance of phosphatase-negative and -positive clones growing on indicator media. Download FIG S1, PDF file, 0.02 MB. Copyright © 2019 Castillo Villamizar et al. 2019 Castillo Villamizar et al. This content is distributed under the terms of the Creative Commons Attribution 4.0 International license.

10.1128/mSphere.00167-19.2 FIG S2 Protein sequence similarities of deduced gene products encoded by the insert of pLP05. Download FIG S2, PDF file, 0.2 MB. Copyright © 2019 Castillo Villamizar et al. 2019 Castillo Villamizar et al. This content is distributed under the terms of the Creative Commons Attribution 4.0 International license.

10.1128/mSphere.00167-19.3 FIG S3 Protein sequence similarities of deduced gene products encoded by the insert of pLP12. Download FIG S3, PDF file, 0.2 MB. Copyright © 2019 Castillo Villamizar et al. 2019 Castillo Villamizar et al. This content is distributed under the terms of the Creative Commons Attribution 4.0 International license.

### The taxonomic classification of the complete inserts indicated that both inserts are of bacterial origin.

**FIG 1:**
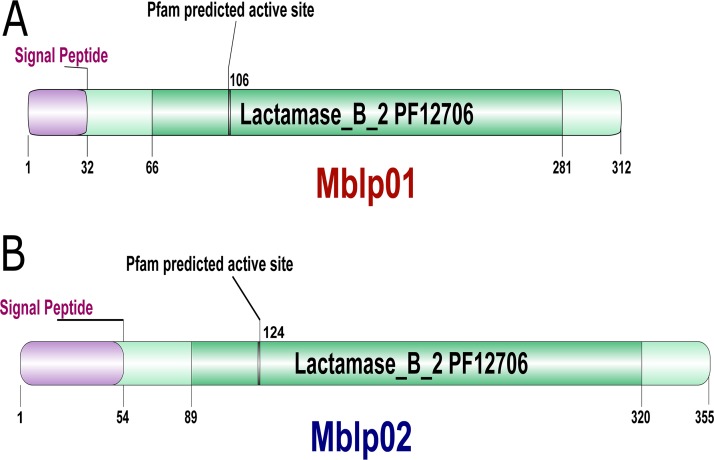
Domain organizations of (A) Mblp01 and (B) Mblp02.

The insert sequence of pLP05 is affiliated with the phylum *Proteobacteria* and that of pLP12 with *Acidobacteria* (see Fig. S4 in the supplemental material). The genes encoding the predicted MBL fold, *mblp01* (pLP05) and *mblp02* (pLP12), respectively, encode polypeptides of 312 and 355 amino acids with estimated molecular masses of 33 and 38 kDa, respectively. The sequence comparison between Mblp01 and Mblp02 showed that both proteins are significantly dissimilar without clear shared conserved regions and have only 29% sequence identity between them. Signal peptides were predicted for both products, suggesting an extracellular or periplasmic function for Mblp01 and Mblp02. The search against the Pfam database assigned both proteins to the lactamase B2 family (PF12706) with E values of 6.3e−24, and 1.4e−16 for Mblp01 and Mblp02, respectively. The domain organizations for both proteins are very similar. The graphic representation of the domain organization according to the Pfam analysis is shown in Fig. 1. The protein sequence analysis against the InterPro database showed that each of these gene products carries signatures of the metallo-β-lactamase domain (IPR001279). Nevertheless, neither of the two proteins could be assigned to any specific family during this analysis, but the signature of the homologous superfamily RNase Z/hydroxyacylglutathione hydrolase-like (IPR036866) was present in both sequences. The sequence similarity searches showed that Mblp01 and Mblp02 exhibited 61% amino acid sequence identity to a hypothetical protein associated with the phylum *Verrucomicrobia* (PYI90218.1) and 51% to a metallo-hydrolase from a *Blastomonas* sp. (WP_054133775.1), respectively. In addition, we performed a search against metagenome databases in order to find the closest homologues of Mblp01 and Mblp02 in environmental samples. Screening of the metagenome-derived protein database from the NCBI (env_nr) yielded sequence identities of 39% (Mblp01) and 49% (Mblp02) to hypothetical proteins derived from marine metagenomes (data not shown). The search against the metagenomic EMBL-EBI database showed a hit (MGYS00000776) with 71.6% sequence identity to Mblp01. MGYS00000776 is derived from a soil metagenome obtained from a Brazilian soil. In the case of Mblp02, the best hit (49% sequence identity) was to the sequence MGYP000565410107, which originated from a geothermal spring water metagenome from India (18).

10.1128/mSphere.00167-19.4 FIG S4 Krona chart showing the taxonomic affiliation of pLP05 and pLP12 inserts determined by Kaiju 1.6.3. Download FIG S4, PDF file, 1.0 MB. Copyright © 2019 Castillo Villamizar et al. 2019 Castillo Villamizar et al. This content is distributed under the terms of the Creative Commons Attribution 4.0 International license.

### Phylogenetic positioning of the new MBL representatives Mblp01 and Mblp02.

**FIG 2:**
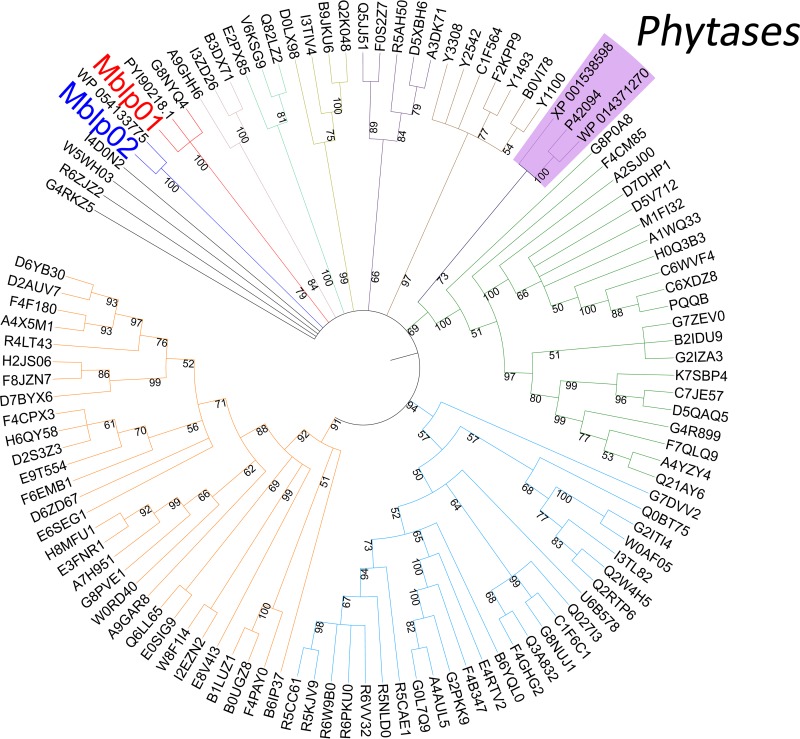
Neighbor-joining phylogenetic tree showing the positions of Mblp01 and Mblp02. The numbers at the nodes indicate levels of bootstrap support (range from 0 to 100) and were based on 1,000 replicates. Names refer to the corresponding UniProtKB/GenBank codes of the extended data in Data Set S1.

To elucidate the relationship between Mblp01 and Mblp02 and other lactamases of the B2 family and classic phytase representatives, we performed a phylogenetic analysis. Sequences of the Pfam seed group that belong to the B2 subclass (PF21706), in addition, representatives of the classical phytases from bacteria (*Blastomonas* sp. and *Bacillus* sp.) and fungi (Histoplasma capsulatum) were included in the analysis. Mblp01 and Mblp02 have the presence of the MBL fold in common, but differ largely between them in sequence composition. Therefore, Mblp01 and Mblp02 grouped separately in the generated phylogenetic tree (Fig. 2; see Data Set S1 in the supplemental material). Mblp01 formed a small cluster with a B2 family MBL of Granulicella mallensis (G8NYQ4) and with its closest BLAST hit, the MBL from *Verrucomicrobia* sp. strain (PYI90218.1). Mblp02 does not group closely with any of the seed MBLs representatives or the phytases but clusters with its closest BLAST hit, WP_054133775.1, from *Blastomonas* sp.

10.1128/mSphere.00167-19.8 DATA SET S1 UniProt/GenBank codes, related proteins, genes, and organisms represented by the sequences used for the construction of the phylogenetic tree depicted in Fig. 2. Download Data Set S1, XLS file, 0.02 MB. Copyright © 2019 Castillo Villamizar et al. 2019 Castillo Villamizar et al. This content is distributed under the terms of the Creative Commons Attribution 4.0 International license.

### Mblp01 and Mblp02 share the same structural analogue: a ZipD protein.

To determine the closest structural relatives, we predicted three-dimensional (3D) models of Mblp01 and Mblp02. This analysis was performed by using the I-TASSER interface (19, 20). The best predicted models of the candidates Mblp01 and Mblp02 showed confidence score (C-score) values of −0.75 and −1.70, respectively (see Fig. S5 in the supplemental material). For both proteins, the predicted models were based on the same Protein Data Bank (PDB) entry (2CBN) (21). This entry corresponds to the crystal structure of a zinc phosphodiesterase (ZipD) from E. coli, which is involved in the tRNA maturation process. As the native structures of Mblp01and Mblp02 are not known, the quality of the modeling prediction was determined by calculating the distance between the predicted models and published native structures. In our case, I-TASSER predicted the quality of the model by calculating the template modeling score (TM-score). TM-scores of 0.783 and 0.697 were calculated for Mblp01 and Mblp02, respectively, indicating a similar structure of the candidate proteins and the reference protein 2CBN.

10.1128/mSphere.00167-19.5 FIG S5 Predicted structures of (A) Mblp01 and (B) Mblp02 by I-TASSER. The figure shows the best model of each enzyme (C-scores of −0.75 and −1.70, respectively). Download FIG S5, PDF file, 2.6 MB. Copyright © 2019 Castillo Villamizar et al. 2019 Castillo Villamizar et al. This content is distributed under the terms of the Creative Commons Attribution 4.0 International license.

### Enzymatic properties of the first reported MBL representatives showing phytase activity.

**FIG 3:**
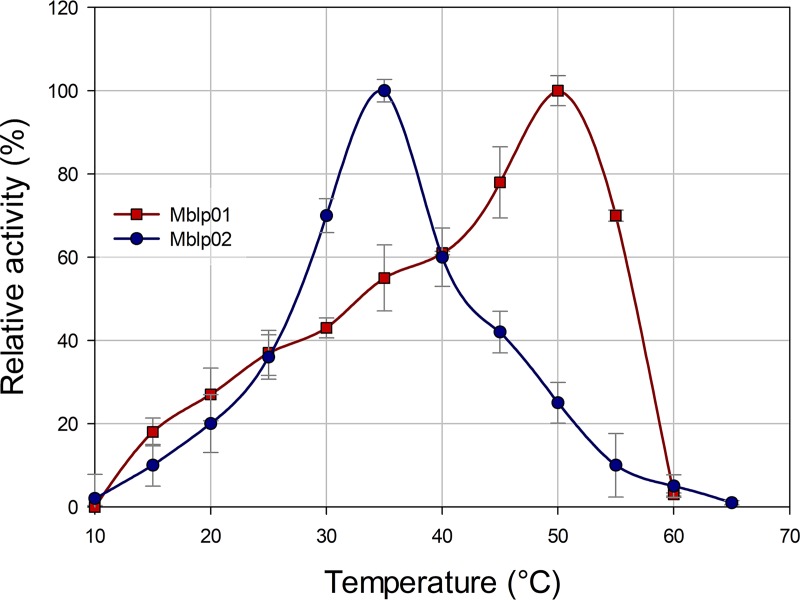
Effect of temperature on the activity of Mblp01 and Mblp02. All measurements were performed in triplicate using the phytase standard assay at temperatures between 10 and 65°C. A relative activity of 100% represented 1.92 ± 0.034 and 1.51 ± 0.069 U/mg for Mblp01 and Mblp02, respectively.

The complete forms Mblp01 and Mblp02 were purified by using a combination of affinity chromatography and size exclusion ultrafiltration. The maximum activities of purified Mblp01 and Mblp02 with phytic acid as the substrate under standard reaction conditions were 50 and 35°C, respectively (Fig. 3).

**FIG 4:**
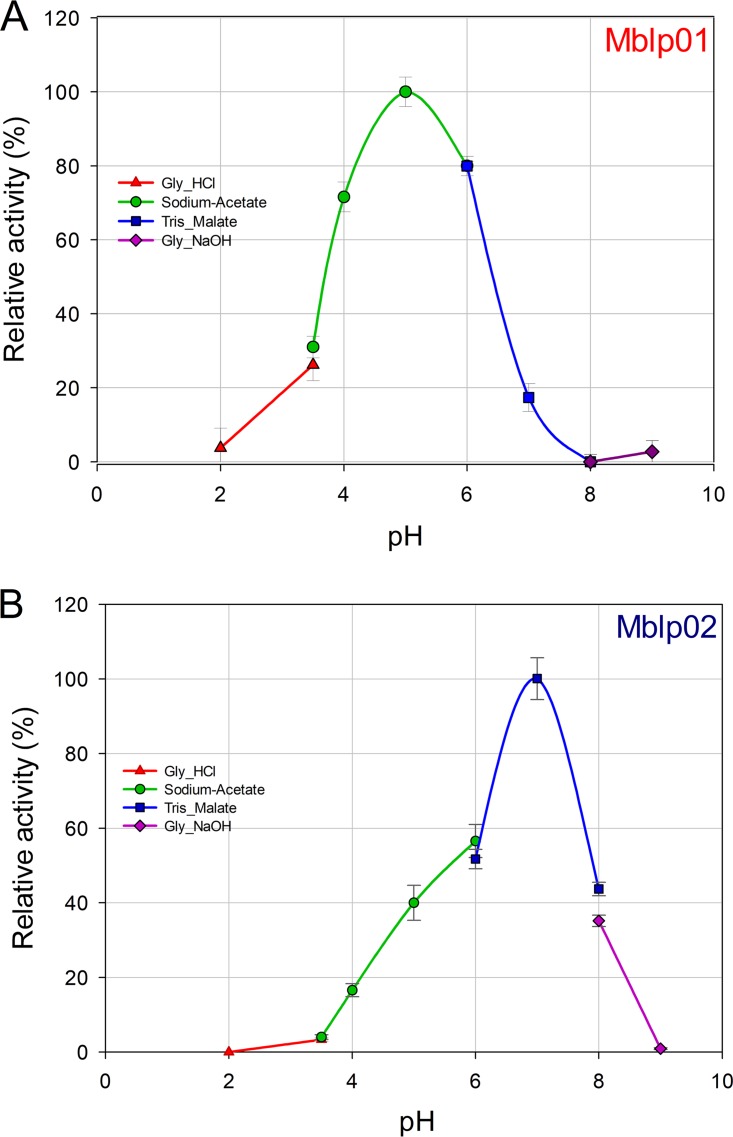
Effect of pH on activity of (A) Mblp01 and (B) Mblp02. The measurements were performed with different buffer systems according to the phytase standard assay at the optimal temperature of each protein. The average from triplicate experiments with the mean deviation is presented. A relative phytase activity of 100% represented 1.78 ± 0.016 and 2.1 ± 0.031 U/mg for Mblp01 and Mblp02, respectively.

**FIG 5:**
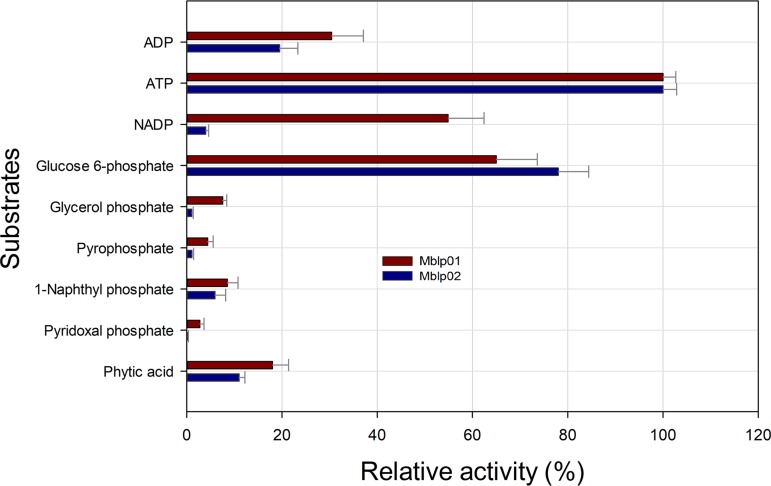
Substrate specificity of Mblp01 and Mblp02. Specific activities corresponding to 100% of activity with ATP as the substrate were 1.77 ± 0.019 and 2.32 ± 0.021 U/mg, respectively. All measurements were performed in triplicate and under optimal pH and temperature conditions for each enzyme.

Mblp01 did not show activity at temperatures below 10 and above 60°C. The activity of Mblp02 increased continually from 10 to 35°C. At higher temperatures, the activity of the enzyme decreased rapidly and was not detectable at 65°C. Dependence of phytase activity on pH was determined in the pH range from 2 to 9 at the optimal temperature of each enzyme activity (Fig. 4). Mblp01 showed activity from pH 2 to 7 and Mblp02 from 3.6 to 8. In the case of Mblp01, more than 70% activity was retained between pH 4 and 6, with maximum activity at pH 5. Mblp02 showed a single narrow activity peak at pH 7.0. Enzymatic activities at pH lower or higher than 7.0 dropped to 60 and 40%, respectively. The ability of Mblp01 and Mblp02 to hydrolyze different phosphorylated compounds was determined under the respective optimal pH and temperature of enzyme activity. Mblp01 showed activity with all tested substrates: Mblp02 could act on most of the substrates, but no activity was seen with pyridoxal phosphate. A main difference of both enzymes is their preference for NADP^+^, whereas Mblp01 possess a relatively high relative activity toward NADP^+^ (55%), Mblp02 has a low relative activity in the presence of this substrate (4%). For both enzymes, the substrates ATP and glucose 6-phosphate yielded the highest activities (Fig. 5). With phytate as the substrate, Mblp01 and Mblp02, respectively, showed 18% ± 3.1% and 11% ± 1.2% activity, relative to the activity measured using ATP, respectively (Fig. 5). The kinetic parameters of both proteins were determined using the purified protein and phytic acid as the substrate. *K_m_* values of Mblp01 and Mblp02 were 1.63 ± 0.031 and 0.4 ± 0.03 mM, respectively. The catalytic efficiencies *k_cat_*/*K_m_* were 159 ± 12 and 367 ± 19 mM^−1^ min^−1^, respectively. The effects of various additives on Mblp01 and Mblp02 enzyme activities are summarized in Fig. S6 and Table S1 in the supplemental material. None of the tested additives enhanced the activity of Mblp01 and Mblp02. Of the evaluated cations, only concentrations higher than 0.5 mM Cu^2+^ and Fe^2+^ showed a significant inhibitory effect on the activity of both enzymes. The presence of SDS and dithiothreitol (DTT) reduced the enzyme activity of both enzymes below 15%.

10.1128/mSphere.00167-19.6 FIG S6 Effect of metal ions on the phytase activity of Mblp01 and Mblp02. The 100% relative activities at concentrations of 1, 0.5 and 0.1 mM are 2.38, 2.61, and 2.77 for Mblp01 and 1.91, 2.03, and 1.94 U/mg for Mblp02, respectively. Download FIG S6, PDF file, 0.04 MB. Copyright © 2019 Castillo Villamizar et al. 2019 Castillo Villamizar et al. This content is distributed under the terms of the Creative Commons Attribution 4.0 International license.

10.1128/mSphere.00167-19.7 TABLE S1 Effect of additives (1 mM) on Mblp01 and Mblp02 activity. The enzyme activities with phytate as the substrate and without any added inhibitor at 2.31 (Mblp01) and 1.79 (Mblp02) U/mg were taken as 100% activity. Values are given as the means from three experiments ± standard deviations. Download Table S1, PDF file, 0.05 MB. Copyright © 2019 Castillo Villamizar et al. 2019 Castillo Villamizar et al. This content is distributed under the terms of the Creative Commons Attribution 4.0 International license.

### Resistance toward β-lactam antibiotics.

Taking into account the promiscuous characteristics of MBLs, the phylogenetic positioning of Mblp01 and Mblp02, and the evolutionary relationship between phosphatases and enzymes that can degrade β-lactam antibiotics (22, 23), the ability of *mblp01* and *mblp02* to confer resistance toward β-lactam antibiotics was tested. For this purpose, MICs and inhibition zones (halos) in the presence of different β-lactam antibiotics were determined. To determine if *mblp01* and *mblp02* confer any level of antibiotic resistance, the host strains E. coli and Shimwellia blattae (formerly Escherichia blattae) (24, 25) harboring the above-generated recombinant plasmids pBAD202-*mblp01* and pBAD202-*mblp02* were evaluated. E. coli was selected as suitable model because the antibiotic resistance profiles for this type of bacteria are well known. *S blattae* was selected because of its close phylogenetic relationship with *E coli* (24), providing the opportunity to compare the effects in two cell lines. Strains carrying the cloning vector without insert were used as controls (Table 1). E. coli and *S. blattae* strains harboring *mblp01* or *mblp02* were less sensitive toward most of the evaluated antibiotics, such as ampicillin and amoxicillin, than the control (Table 1). In addition, both proteins process the tested antibiotics differently. Mblp02 increased on a larger scale the resistance of E. coli and *S. blattae* against ceftazidime than Mblp01. Thus, a β-lactamase activity was indicated under the tested conditions and suggested that MblP01 and MblP02 are promiscuous enzymes.

**TABLE 1:** Sensitivity against β-lactam antibiotics of E. coli and *S. blattae* strains harboring the *mblp01-* and *mblp02-*containing plasmids (pBAD202*-mblp01* or pBAD202-*mblp02*) and, as a control, the cloning vector without insert (pBAD202)

Strain construct	MIC (μg/ml)^*a*^				HIZ (mm)^*b*^	
	Ampicillin	Imipenem	Ceftazidime	Amoxicillin	Doripenem (10 μg)	Cefaclor (30 μg)
E. coli						
pBAD202	1.2 ± 0.5	0.8 ± 0.3	0.2 ± 0.1	5.3 ± 3.1	14.5 ± 1.6	12.5 ± 1.9
pBAD202-*mblp01*	6.7 ± 2.1	1.2 ± 0.5	1.3 ± 0.5	13.3 ± 4.1	9.7 ± 0.8	9.5 ± 0.9
pBAD202-*mblp02*	8.0 ± 0.0	3.3 ± 1.0	5.3 ± 2.1	13.3 ± 4.1	8.8 ± 0.8	7.2 ± 0.8
*S. blattae*						
pBAD202	1.5 ± 0.8	0.8 ± 0.3	0.3 ± 0.2	3.7 ± 0.8	13 ± 0.9	10 ± 0.9
pBAD202-*mblp01*	3.3 ± 1.0	0.5 ± 0.0	0.5 ± 0.4	6.0 ± 0.0	7.7 ± 1.4	8.3 ± 0.5
pBAD202-*mblp02*	2.7 ± 1.0	1.7 ± 0.5	0.7 ± 0.3	3.3 ± 1.0	7.5 ± 1.6	6.5 ± 1.2

a Shown are MICs of selected β-lactam antibiotics.

b HIZ, diameter of inhibition zone (halo) in relation to each utilized antibiotic based on the antibiotic concentration on the test disc. Values are the average from four experiments.

## DISCUSSION

Certain types of phytases are responsible for phytate breakdown during seed germination to make phosphate and *myo*-inositol available for plant nutrition and development (26). The microbial production of extracellular phytase improves phosphate availability in plant roots and overcomes phosphate starvation for example in Phaseolus vulgaris (27, 28). In addition, phytases have been reported to be involved in signal transduction, cell division, and microbial pathogenesis (11, 12, 29). Despite their broad relevance, phytase research has been mainly focused on improvement of enzymes for use as animal feed additives. Very little is known about diversity of phytases or their roles *in vivo* (30). Phosphatases are in general enzymes that hydrolyze a broad spectrum of phosphorylated compounds, including phytate. However, reported phytase activity is limited to a few protein types, as mentioned above (7). One limiting factor for finding new types of proteins or catalytic domains associated with phytase activity is the almost exclusive usage of individual microorganisms for the isolation and characterization of this type of enzyme (14, 31).

Our analysis showed that the metagenome-derived enzymes Mblp01 and Mblp02 carry the MBL catalytic domain and originate from bacteria. Nevertheless, the amino acid sequence identities toward known proteins are low: 51% toward a metallo-hydrolase from *Blastomonas* sp. and 61% toward a hypothetical protein from *Verrucomicrobia*. This is a good indication of the novelty of both enzymes. To the best of our knowledge, similar proteins were not characterized before. The genes *mblp01* and *mblp02* were derived from forest soil metagenomes. Searches of metagenomes revealed that the product Mblp01 is similar to a protein deduced from a forest soil metagenome in Brazil, indicating that enzymes with similar characteristics might be present in other forest soils.

The sequences of Mblp01 and Mblp02 clustered differently in the constructed phylogenetic tree. Mblp01 is part of a small cluster together with an uncharacterized MBL protein (G8NYQ4). G8NYQ4 is derived from a genome sequence of Granulicella mallensis, which was originally isolated from tundra soil of northwestern Finland (32). Mblp02 clusters with the sequence WP_054133775.1 from *Blastomonas* sp., which is also a type of bacteria originally isolated from soil samples (33). All this suggest that Mblp01 and Mblp02 are indeed proteins associated with a process of soil bacteria. Several representatives of protein phytases were integrated in the general topology of the cluster. Nevertheless, none of the included phytases nor Mblp02 forms a specific group with the known MBLs (Fig. 2). The evolutionary link between MLBs and phosphatases has been reported, but is not fully clear (23). Chakraborty et al. in 2012 described the likelihood of the presence of a class B2 MBL-like scaffold in a cold active alkaline phosphatase from *Vibrio* sp. (22). The prediction was verified by the inhibition of the phosphatase activity by imipenem. The results suggested a natural evolution of the alkaline phosphatases to acquire true metallo-β-lactamase activity (22).

The predicted models of Mblp01 and Mblp02 provided the first insights into the structure of these proteins (Fig. S5). The models are supported by the calculated C- and TM-scores and show a remarkable relationship of both enzymes with a zinc phosphodiesterase encoded by the gene *elaC* from E. coli. ElaC harbors the MBL domain and possesses phosphodiesterase activity. Additionally, it is affiliated with the tRNase Z family and acts as a clamp in tRNA binding (21).

Characterization of phytases has shown that these enzymes exhibit a wide range of features. It has been reported that phytases are usually most active within temperature ranges of 45 to 60°C (31). However, these high ranges might be due to the fact that most research focused on thermophilic phytases from individual microorganisms, which can be used in industrial applications. With the identification of metagenome-derived phytases, the temperature range of the optimal phytase activity changed. PhyRC001, a metagenome-derived phytase from red rice, showed optimal activity at 30°C (15). The Mblp02 activity optimum was 35°C. Similarly, another recently described soil metagenome-originated phytase (Pho07) showed highest activities at lower temperatures (25 to 30°C) (14). The reported pH range of phytase activity also varies (2.2 to 8.0), whereby phytases of bacterial origin revealed optimal activities between pH 6.5 and 7.5. A similar pH range was recorded for the optimal activities of Mblp01 and Mblp02, which according to our analysis are of bacterial origin. Nevertheless, other soil metagenome-derived bacterial phytases (i.e., Pho07) exhibited a pH optimum of 4.0 (14).

MBLs and phosphatases are both considered promiscuous enzymes with respect to substrate spectrum or, in some cases, catalytic mechanism (34, 35). It has been estimated that MBLs can catalyze on average 1.5 reactions. Moreover, directed evolution experiments have shown that with a few mutations, the β-lactamases NDM1 and VIM2 can be converted to enzymes with a promiscuous phosphonate monoester hydrolase activity (36). A similar explanation might be valid for the promiscuous Mblp01 and Mblp02 with respect to the additional indicated β-lactamase activity of both enzymes. The tested host strains of E. coli and *S. blattae* carrying and expressing *mblp01* and *mblp02* show less susceptibility against the tested β-lactam antibiotics than the control strains. It has been previously suggested that the MBL superfamily could have evolved from a common ancestor via promiscuous enzymes with a connected catalytic landscape (16, 36). Some zones of sequence space may overlap multiple catalytic landscapes, including sequences of enzymes that can catalyze more than one activity (catalytic promiscuity). The connections between different catalytic landscapes amend enzymes to evolve and develop new functions (37). Some of the new enzymatic functions in the MBL superfamily such as phosphodiesterase activity evolved rapidly from the current diversity of enzymes with promiscuous activities. Other MBLs have evolved recently on the basis of the appearance of substrates that were not available a few years ago. A good example are phosphotriesterases, which hydrolyze organophosphate pesticides. This type of enzyme evolved and developed activity toward a substrate that did not exist 60 to 80 years ago (36). The β-lactamase activity was invented several times during evolution in independent ways. It has been hypothesized that promiscuous activities provide an immediate evolutionary advantage against β-lactam antibiotics in an environment in which antibiotics are present (36).

Relatively few organisms use phytate as the sole P source. Most reported phosphatases with phytase activity are nonspecific phosphatases (30). The *K_m_* values of Mblp01 and Mblp02 are in the same range of other reported environmental phosphatases with phytase activity (e.g., Pho07 and Pho16B), suggesting low-affinity values for phytate in enzymes with a broad substrate range (14, 38). The catalytic efficiencies toward phytate of Mblp01 and Mblp02 are significantly lower than the catalytic efficiencies of other reported phytases derived from cultured single microorganisms (39–41). The studies with the additives revealed that SDS and DTT exhibited deleterious effects on activity of Mblp01 and Mblp02. In the presence of SDS, the activity of both enzymes was strongly reduced or not detectable. SDS has been reported to be a strong inhibitor of phytases (42). Anionic detergents bind to proteins and induce structural changes that affect the protein’s stability, functionality, and solubility (43). DTT had also a strong inhibitory effect on the phytase activity of Mblp01 and Mblp02. The same effect was observed for the environmental phytase Pho16B (38). DTT acts as chelator of metal ions essential for enzyme activity (44). The cation Cu^2+^ exhibited an inhibitory effect on both enzymes (Fig. S6). It has been reported that copper ions directly inactivate other proteins of the metallo-β-lactamase superfamily and also phytases. However, the mechanism leading to this inactivation remains unknown (45).

In conclusion, the applied function-driven metagenomic approach resulted in identification of two representatives of a new type of phytate-degrading enzymes exhibiting an MBL domain structure. This study shows that MBLs are potentially involved in a previously unreported process, recovering valuable P from phytate. Our data improve the knowledge on the diversity of phytate-degrading enzymes, which is required to gain insights into the relationships among these enzymes. This knowledge could help in the future to design and engineer superior biocatalysts as well as improve our capabilities to solve problems such as the P scarcity and the proliferation of antibiotic-resistant bacteria.

## MATERIALS AND METHODS

### Sampling, metagenomic library construction, and clone selection.

The genes encoding Mblp01 and Mblp02 originate from two soil samples, SEW46 and HEW30, respectively. The pH values were 3.29 for SEW46 and 3.86 for HEW30 (46). Both samples were collected from A horizons (topsoil) from beech forest sites in Germany. SEW46 was collected within the Schorfheide-Chorin biosphere reserve, while the HEW30 sample was collected within Hainich National Park. Collection of the samples was performed as previously described by Kaiser et al. (47). Approximately 2.5 g of soil was used for total DNA extraction by employing the PowerSoil DNA isolation kit (MoBio Laboratories, Carlsbad, CA). The metagenomic libraries were generated using the method described by Nacke et al. (46) and constructed and screened by Castillo Villamizar et al. (14, 46). The metagenomic libraries SEW46 and HEW30 were composed of 38,122 and 53,460 clones, respectively (14). Libraries were constructed by using the plasmid pCR-XL-TOPO (Invitrogen GmbH, Karlsruhe, Germany). This vector allows the cloning of long PCR products up to 13 kb and possesses a high transformation efficiency. In addition, pCR-XL-TOPO is IPTG (isopropyl-β-d-thiogalactopyranoside) inducible, allowing a simple control of the cloned genes into the library. The library-bearing E. coli clones were screened by using modified minimal Sperber minimal medium (16 g/liter agar, 10 g/liter glucose, 500 mg/liter yeast extract, 100 mg/liter CaCl_2_, and 250 mg/liter MgSO_4_). In order to induce phytase activity, phytate (2.5 g/liter) was used as the phosphorus source and 25 μg/ml of 5-bromo-4-chloro-3-indolyl phosphate (BCIP) as the indicator. Clones with phosphatase/phytase activity turned from white to dark blue within 48 h (48, 49).

### Sequence data analysis.

The insert sequences of plasmids pLP05 and pLP12, derived from the libraries SEW46 and HEW30, respectively, were sequenced and analyzed. Initially, the taxonomic classification of the complete DNA inserts of pLP05 and pLP12 was performed by using the software Kaiju (50). Next, open reading frame (ORF) prediction was performed using the ORF finder tool provided by the National Center for Biotechnology Information (NCBI) and the ARTEMIS program (51, 52). The results were verified manually by using criteria such as the presence of a ribosome-binding site, GC frame plot analysis, and similarity to known genes.

Amino acid sequences deduced from the *mblp01* and *mblp02* gene products were examined for similarities to known protein families and domains by performing searches against the Pfam, InterPro, and NCBI collections. Signal peptide prediction was performed using SIGNALP 5.0 (53–55). Mblp01 and Mblp02 were also analyzed by using the Basic Local Alignment Search for proteins (blastp). Two NCBI databases—nonredundant sequences (nr) and metagenomic proteins (env_nr)—were employed. An additional search was performed against the metagenomic platform of the European Institute of Biotechnology (EMBL-EBI) (54, 56). Multiple sequence alignments of Mblp01 and Mblp02 and related MBLs were performed using MUSCLE (57). Evolutionary analyses were conducted in MEGA 7 using the neighbor-joining method (58). The bootstrap consensus tree was inferred from 1,000 replicates. The evolutionary distances were computed using the number of differences method. The analysis involved 113 amino acid sequences (59) and a total of 607 positions in the final data set. Branches with bootstrap values below 50% were collapsed. The tree was visualized using iTOL v3 (60). A prediction of the tertiary structure of the proteins Mblp01 and Mblp02 was performed by employing the I-TASSER platform (20). The quality of models generated using I-TASSER is based on two major criteria: the confidence score (C-score) and the template modeling score (TM-score) (19). I-TASSER generated five models for each protein. The models were ranked based on the C-score. The C-scores are calculated on the basis of the statistical significance of the threading profile-profile alignment, as well as structure convergence of the assembly simulations. The C-scores ranged from −5 to 2. A high C-score value indicates a model with higher confidence (19). The TM-score addresses the structural similarity of two protein models by measuring the global fold similarity. TM-score is less sensitive to local structural variations, and its magnitude for random structure pairs is length independent. The TM-score has a value range of 0 to 1, whereby 1 indicates a perfect match between two structures (19, 61). By calculating the TM-score, the structural similarity between the predicted models of Mblp01 and Mblp02 and other published determined structures is estimated. Values close to 0.5 indicate a model of correct topology. In this study, the models with the highest C-score were selected as the best predicted optimized 3D modeling structure.

### Protein expression and purification.

To facilitate expression and purification, *mblp01* and *mblp02* were cloned into plasmid pBAD202/d-TOPO according to the instructions of the manufacturer (Thermo Fisher Scientific GmbH, Schwerte, Germany). In this way, sequences encoding the His_6_ and thioredoxin tags were added to the N terminus of the produced proteins during cloning. As a control, a noncoding DNA region was also cloned in pBAD202/d-TOPO. The fidelity of the constructs was confirmed by Sanger sequencing. The generated constructs were used to transform Escherichia coli LMG194. Transformants were grown on Sperber screening medium supplemented with 0.2% arabinose. Only the clones carrying a recombinant plasmid harboring *mblp01* or *mblp02* showed phosphatase/phytase activity on indicator agar after transformation.

The expression plasmids containing *mblp01* or *mblp02* (pBAD202-*mblp01* and pBAD202-*mblp02*, respectively) were transformed into Escherichia coli LMG194. Subsequently, the recombinant E. coli strains were grown on Luria-Bertani (LB) agar plates supplemented with kanamycin (50 μg/μl) and incubated at 37°C. A single colony of each construct was used to inoculate 1 liter of M9 minimal salts medium (62) containing 50 μg/μl kanamycin and 2% glycerol. The culture was incubated using a New Brunswick Innova 44 incubator-shaker (Eppendorf AG, Hamburg, Germany) with shaking (90 rpm) at 37°C. Protein expression was induced at an optical density at 600 nm (OD_600_) of 0.6 using l-arabinose (final concentration, 0.2%). Cells were harvested after 5 h of incubation by centrifugation for 30 min at 4°C and 8,000 rpm (Sorvall RC6 centrifuge, rotor SLA 3000; Thermo Fisher Scientific). The resulting cell pellets were suspended in 10 ml of 50 mM HEPES buffer containing 250 mM NaCl and 0.5 mM ZnSO_4_. Mechanical cell disruption was performed using a French press (1.38 × 10^8^ Pa; Thermo Fisher Scientific). Subsequently, the extract was cleared by centrifugation for 0.5 h at 4°C and 15,000 rpm (Sorvall RC6 centrifuge with rotor SS 35; Thermo Fisher Scientific). The crude extract was filtered using filters with a pore size of 0.45 μm and then with 0.2-μm-pore filters (Sarstedt, Nümbrecht, Germany). In order to purify the His_6_-tagged protein, the Protino Ni-TED 2000 purification kit was used as recommended by the manufacturer (Macherey and Nagel, Düren, Germany) with modifications. The equilibration of the columns and the washing steps were performed with 50 mM HEPES (pH 8.0) containing 250 mM NaCl and 0.5 mM ZnSO_4_. Elution was performed with 50 mM HEPES containing 250 mM NaCl, 250 mM imidazole, and 0.5 mM ZnSO_4_. Buffer exchange and imidazole removal were performed by ultrafiltration using Vivaspin 20 concentrators with an exclusion limit of 30 kDa combined with VS20 diafiltration cups as recommended by the manufacturer (Sartorius AG, Göttingen, Germany). The thioredoxin tag of the proteins was removed by employing the enterokinase cleavage capture kit as recommended by the manufacturer (Merck KGaA, Darmstadt, Germany) with a modified cleavage buffer consisting of 50 mM Tris-HCl (pH 7.4), 50 mM NaCl, 2 mM CaCl_2_, and 0.5 mM ZnSO_4_. Subsequently, ultrafiltration of the protein extract using a Vivaspin concentrator with exclusion limit of 10 kDa was performed.

### Biochemical characterization of Mblp01 and Mblp02.

Phosphatase activity was determined at 355 nm by detecting the release of inorganic phosphorus according to the ammonium molybdate method developed by Heinonen and Lahti with modifications (63, 64). The purified enzyme solution (10 μl) was preincubated for 3 min at 40°C in 380 μl of 50 mM sodium acetate buffer (pH 5). Subsequently, 10 μl of 100 mM phytic acid dipotassium salt (Sigma-Aldrich, Munich, Germany) was added, and the mixture was incubated for 30 min at 40°C. To stop the reaction, 1.5 ml of freshly prepared AAM solution (acetone, 5 N H_2_SO_4_, 10 mM ammonium molybdate) and 100 μl 1 M citric acid were added. Samples were measured against blanks prepared by adding AAM solution prior to the addition of enzyme. The absorbance (355 nm) was measured using the Ultrospec 3300 Pro (Amersham Plc, Little Chalfont, United Kingdom). All measurements were performed in triplicate. To calculate the enzyme activity, a calibration curve was generated in the range of 5 to 600 nmol phosphate. One activity unit represents the release of 1 nmol phosphate per min.

The influence of temperature on enzymatic activity was determined via the above-described standard phytase assay. The enzymatic activity was evaluated in a temperature range of 10 to 65°C by using a temperature-adjusted buffer (50 mM sodium acetate, pH 6). In order to analyze the pH dependence of enzyme activity, the following overlapping buffers were prepared as described by Gomori (65): 50 mM glycine-HCl (pH 2.0, 3.0, and 3.6), sodium acetate (pH 3.6, 4.0, 5.0, and 6.0), Tris-malate (pH 6.0, 7.0, and 8.0), Tris-HCl (pH 8.0 and 9.0), and glycine-NaOH (pH 9.0).

The substrate specificity was determined using the standard phytase assay under the optimal temperature and pH conditions. Nine different substrates comprising ADP, ATP, NADP, glucose-6-phosphate, glycerophosphate, pyridoxal phosphate, pyrophosphate, naphthyl phosphate, and phytic acid were tested using the 10 mM concentration. Furthermore, the effects of cations (Al^2+^, Ca^2+^, Cu^2+^, Co^2+^, Fe^2+^, and Mg^2+^) and the potential inhibitors ethylenediaminetetraacetic acid (EDTA), tungstate, oxalate, sodium dodecyl sulfate (SDS), and dithiothreitol (DTT) (1 mM) were analyzed. Kinetic parameters *K_m_* and *k_cat_/K_m_* for both enzymes were calculated from the Michaelis-Menten equation by using the kinetics module of the program SigmaPlot 12.0 (Systat Software, Inc., San Jose, CA, USA). All measurements were performed under optimal pH and temperature conditions using phytate as the substrate.

In addition to the biochemical characterization of the enzymes Mblp01 and Mblp02, an analysis of the antimicrobial activity of the gene products encoded by *mblp01* and *mblp02* was performed. The MIC for β-lactam antibiotics was determined. Ampicillin, imipenem, ceftazidime, and amoxicillin strips (M.I.C.Evaluator; Oxoid, Basingstoke, UK) were used. Two additional antibiotics—doripenem and cefaclor (Oxoid)—were evaluated by measuring the inhibition zone (halos) around discs containing 10 μg doripenem or 30 μg cefaclor. E. coli TOP10 and Shimwellia blattae DSM 4881 were transformed with the pBAD202-*mblp01* and pBAD202-*mblp02* plasmids. Strains harboring the cloning vector pBAD202 without insert served as a control. All recombinant strains were analyzed in duplicate using the M.I.C.Evaluator system according to the instructions of the manufacturer (Oxoid) with Luria-Bertani (LB) agar containing 50 μg/ml kanamycin and 0.2% arabinose (pH 7.0).

### Data availability.

The sequences of the inserts have been submitted to the National Center for Biotechnology Information (NCBI) under GenBank accession no. MH367836 (pLP05) and MH367837 (pLP12).
