# Supplementary figures and images for: Functional Metagenomics Reveals a New Catalytic Domain, the Metallo-β-Lactamase Superfamily Domain, Associated with Phytase Activity
Source: mSphere. 2019 Jun 19;4(3):e00167-19. doi: 10.1128/mSphere.00167-19 (PMC6584368; doi:10.1128/mSphere.00167-19)

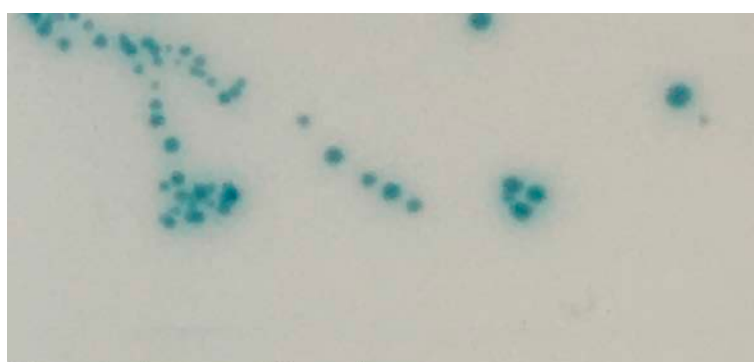

Phosphatase positive clone

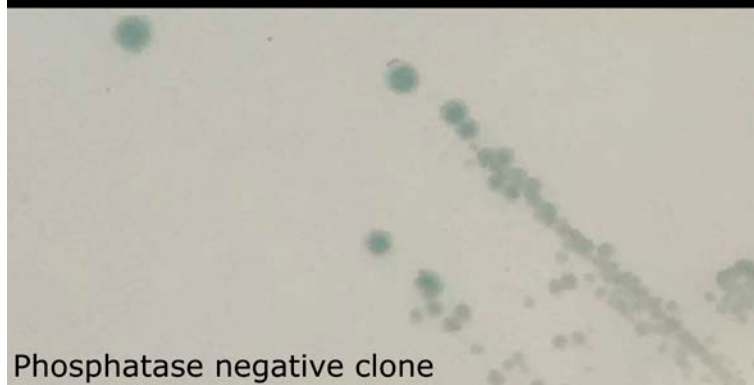

Phosphatase negative clone

Supplement: FIG S1 [file mSphere.00167-19-sf001.pdf]

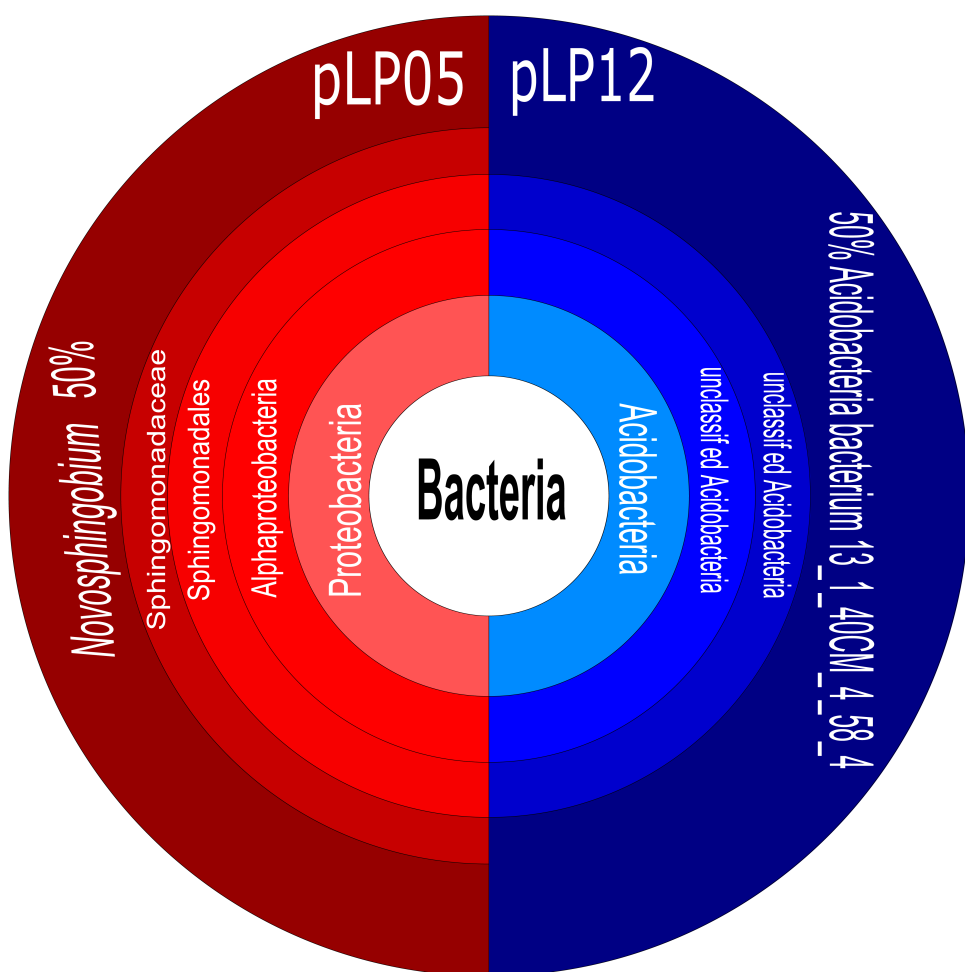

Supplement: FIG S4 [file mSphere.00167-19-sf004.pdf]

*A*

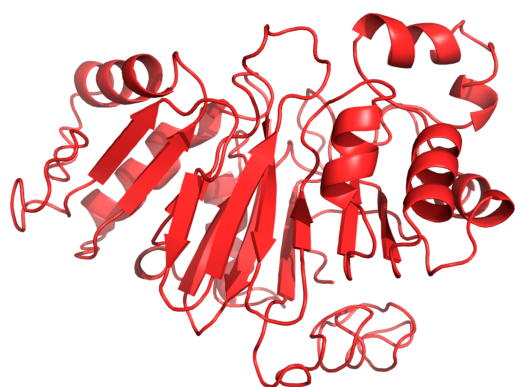

*B*

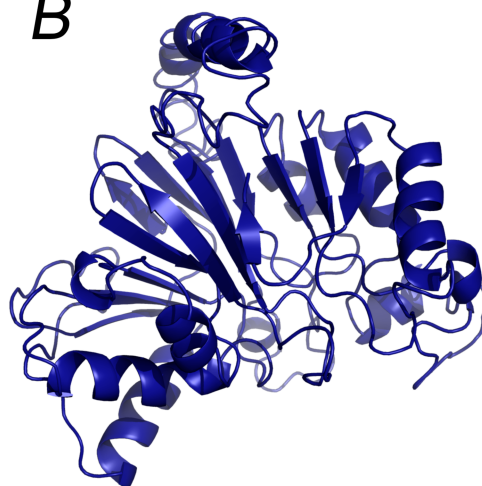

Supplement: FIG S5 [file mSphere.00167-19-sf005.pdf]

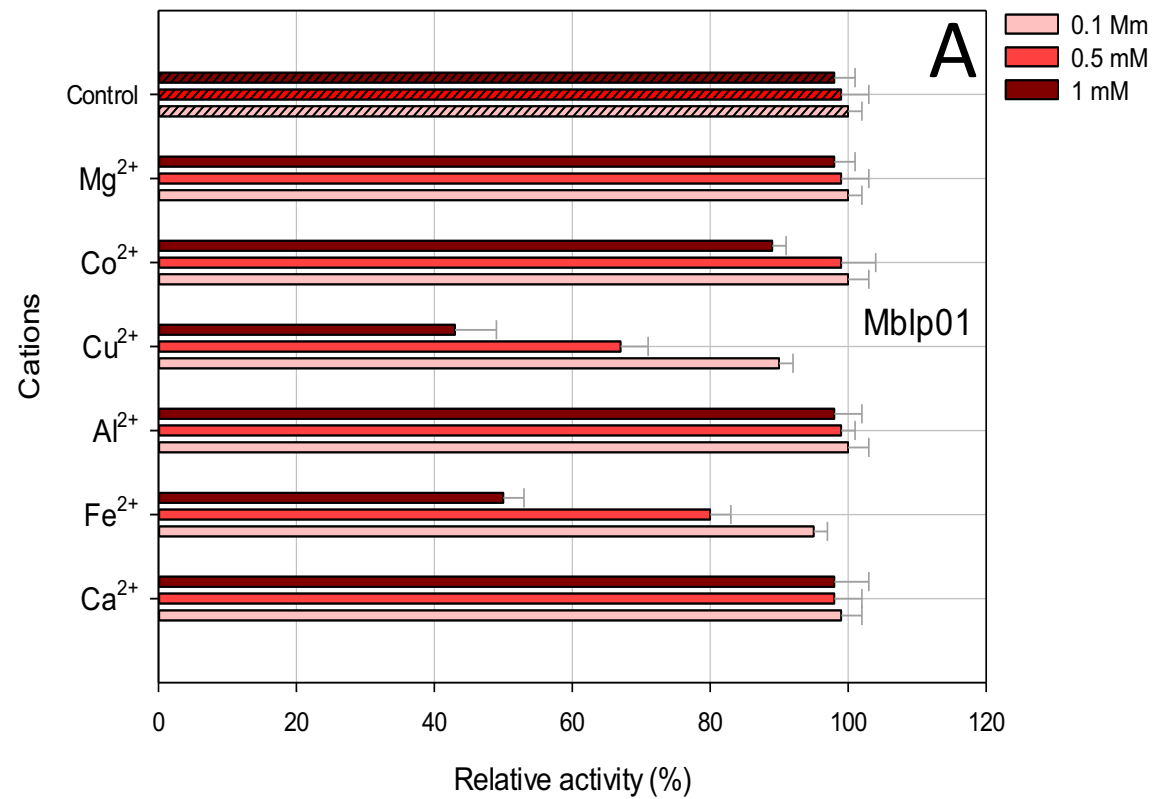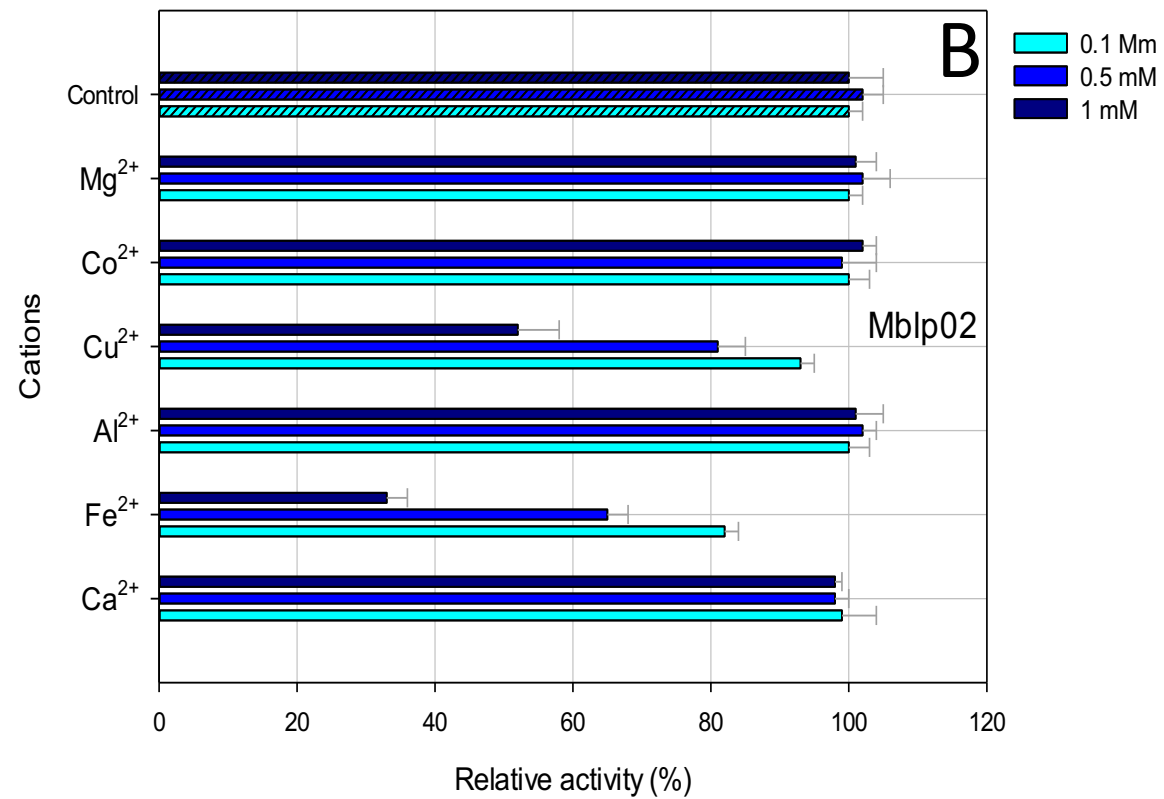

Supplement: FIG S6 [file mSphere.00167-19-sf006.pdf]
