# Supplementary material for: Functional Metagenomics Reveals a New Catalytic Domain, the Metallo-β-Lactamase Superfamily Domain, Associated with Phytase Activity
Source: mSphere. 2019 Jun 19;4(3):e00167-19. doi: 10.1128/mSphere.00167-19 (PMC6584368; doi:10.1128/mSphere.00167-19)
Supplement: FIG S2 [file mSphere.00167-19-sf002.pdf]

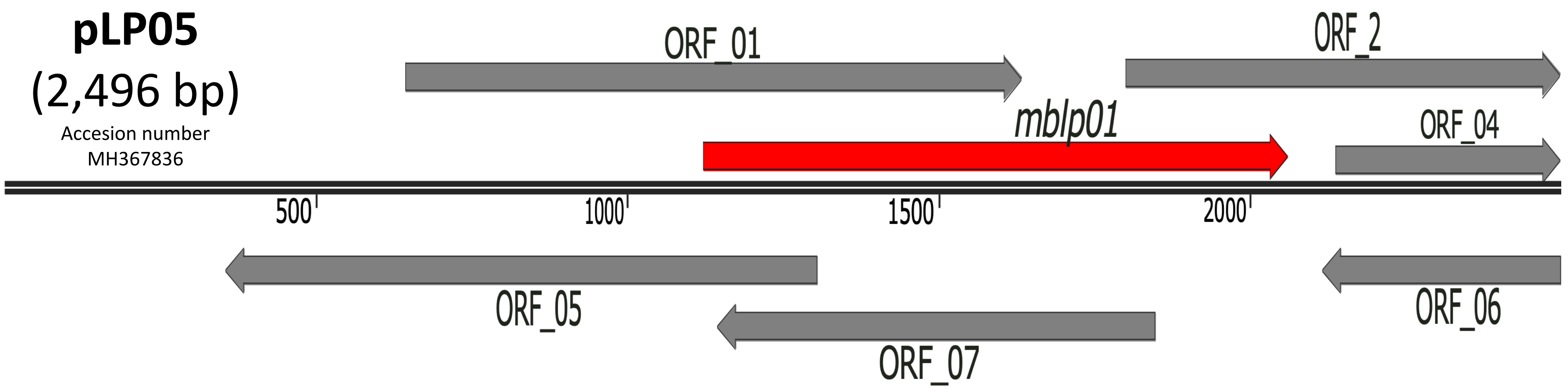

ORF number	Position/strand	SmartBLAST result
ORF_01	644 - 1633 (+)	No SmartBLAST hits found
ORF_02	1801 - 2496 (+)	No SmartBLAST hits found
mblp01	**1122 - 2060 (+)**	**MBL fold metallo-hydrolase (Verrucomicrobia)**
ORF_04	2138 - 2494 (+)	LysR family transcriptional regulator, partial (Acidobacteria)
ORF_05	356 - 1303 (-)	DUF72 domain-containing protein (Acidobacteria)
ORF_06	2117-2496 (-)	Disulfide bond formation protein B (gamma proteobacterium)
ORF_07	1146 - 1847 (-)	No SmartBLAST hits found
