# Supplementary material for: Functional Metagenomics Reveals a New Catalytic Domain, the Metallo-β-Lactamase Superfamily Domain, Associated with Phytase Activity
Source: mSphere. 2019 Jun 19;4(3):e00167-19. doi: 10.1128/mSphere.00167-19 (PMC6584368; doi:10.1128/mSphere.00167-19)
Supplement: FIG S3 [file mSphere.00167-19-sf003.pdf]

**pLP12**

(5,578 bp)

Accesion number
MH367837

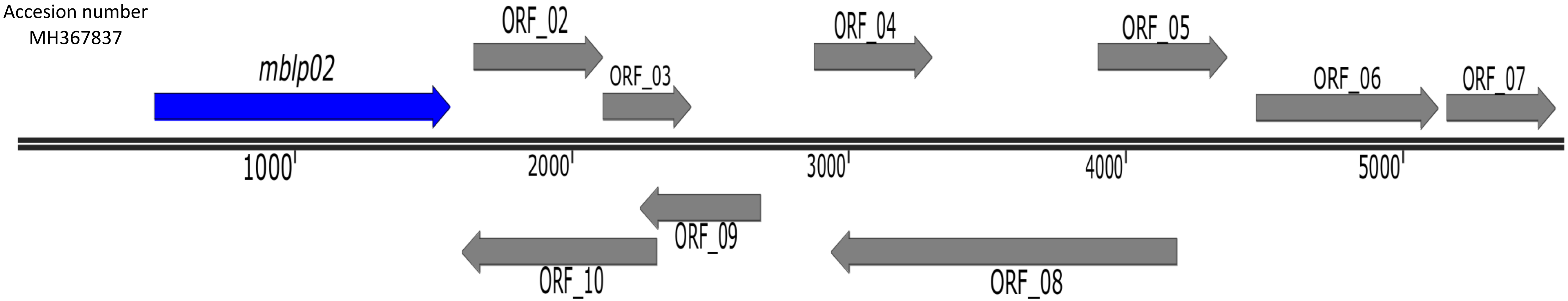


| ORF number | Position/strand | SmartBLAST result |
|---|---|---|
| ***mbpl02*** | **494 - 1561 (+)** | **MBL fold metallo-hydrolase [*Sphingobium*]** |
| ORF_02 | 2113 - 2433 (+) | Plasmid maintenance protein CcdB (*Sulfurirhabdus autotrophica*) |
| ORF_03 | 2876 - 3304 (+) | glycosyltransferase family 2 protein (*Sphingobium* sp. D43FB) |
| ORF_04 | 3902 - 4369 (+) | No SmartBLAST hits found |
| ORF_05 | 4475 - 5131 (+) | serine hydrolase )*Phenylobacterium* sp.) |
| ORF_06 | 5163 - 5555 (+) | No SmartBLAST hits found |
| ORF_07 | 1646 - 2113 (+) | plasmid maintenance protein CcdB (Rhodocyclales) |
| ORF_08 | 2945 - 4189 (-) | No SmartBLAST hits found |
| ORF_09 | 2250 - 2681 (-) | No SmartBLAST hits found |
| ORF_10 | 1609 - 2313 (-) | No SmartBLAST hits found |
