# Supplementary material for: Functional Metagenomics Reveals a New Catalytic Domain, the Metallo-β-Lactamase Superfamily Domain, Associated with Phytase Activity
Source: mSphere. 2019 Jun 19;4(3):e00167-19. doi: 10.1128/mSphere.00167-19 (PMC6584368; doi:10.1128/mSphere.00167-19)
Supplement: TABLE S1 [file mSphere.00167-19-st001.pdf]

Table S1. Effect of additives (1 mM) on Mblp01 and Mblp02 activity. The enzyme activity with phytate as substrate and without any added inhibitor was taken as 100% activity, 2.31 and 1.79 U/mg for Mblp01 and Mblp02, respectively. Values are given as the mean of three experiments ± standard deviations.

**Inhibitors**	**Phytase activity (%)**	
	Mblp01	Mblp02
Control	100 ± 0.83	100 ± 0.69
EDTA	50.38 ± 1.12	62.85 ± 1.46
Wolframate	45.52 ± 2.53	22.05 ± 2.33
Oxalate	69.19 ± 1.98	35.56 ± 3.02
SDS	N.D.	2.92 ± 1.61
DTT	7.21 ± 0.92	14.02 ± 2.51

N.D., not detected.
